# Interocular asymmetry and ocular biometric patterns in pediatric high myopia: implications for early risk stratification

**DOI:** 10.3389/fmed.2026.1841384

**Published:** 2026-05-25

**Authors:** Siqi Zhang, Xi Wang, Zhaoxing Ding, Qi Zhao

**Affiliations:** Department of Ophthalmology, The Second Affiliated Hospital of Dalian Medical University, Dalian, Liaoning, China

**Keywords:** AL/CR, axial length of the eye, children and adolescents, high myopia, myopia prevention and control

## Abstract

**Purpose:**

To describe the clinical and ocular biometric characteristics of high myopia in children and adolescents, analyze the relationship between age and axial length (AL) and axial ratio (AL/CR), identify high-risk structural populations, and evaluate binocular asymmetry and follow-up progression.

**Methods:**

We retrospectively extracted data from the outpatient medical record system of our hospital, including patients whose age at the last follow-up was less than 18 years and whose spherical equivalent (SE) of at least one eye was ≤ −6.00D. The last follow-up data were used as the main analysis, and best corrected visual acuity (BCVA), SE, axial length (AL), and AL/corneal radius (AL/CR) were extracted. Pearson correlation analysis was used to evaluate the correlation between age and AL, AL/CR. In addition, the differences between the left and right eyes of the same patient (| ΔSE|, | ΔAL|) were calculated, and a longitudinal description was made for the subset with repeated biometric records.

**Results:**

A total of 84 patients (47 males and 37 females) were included, with a total of 146 highly myopic eyes. The average spherical equivalent (SE) of highly myopic eyes was −8.87 ± 2.58D; the axial length (AL) was 26.41 ± 1.33 mm (*n* = 118); and the AL/corneal radius (CR) ratio was 3.38 ± 0.19 (*n* = 73). Age was significantly positively correlated with AL (*r* = 0.485, *P* < 0.001), and age was also significantly positively correlated with the AL/CR ratio (*r* = 0.505, *P* < 0.001). Generalized estimating equations (GEE) confirmed that age independently predicted AL (β = 0.200 mm/year, *P* < 0.001) and AL/CR (β = 0.028 per year, *P* < 0.001), and the effect of SE on AL remained significant after adjusting for age (β = −0.239 mm/D, *P* < 0.001). Anisometropia was relatively common in both eyes of the patients, with | ΔSE| ≥ 2.0D accounting for 27.7%. In the follow-up subset (34 eyes), the annual growth rate of AL was approximately 0.224 ± 0.264 mm/year.

**Conclusion:**

Children with high myopia show obvious ocular structural abnormalities and heterogeneous progression. GEE analysis further validated the independent contributions of age and refractive error to axial elongation in this population. AL and AL/CR can be used as key structural indicators for follow-up; unilateral high myopia or significant binocular asymmetry suggests the need for enhanced individualized myopia monitoring and intervention.

## 1 Introduction

In recent years, the global prevalence of myopia has risen sharply, becoming a global public health crisis (1–3). Due to specific social circumstances, China is facing an unprecedented challenge of myopia prevalence. Under the intense educational competition, children in our country have long been burdened with heavy academic loads, excessive exposure to electronic screens, and a severe lack of outdoor activity time (4, 5). Given the severity of the situation, China has officially elevated the prevention and control of myopia among children and adolescents to a national public health strategy. However, under the continuous interplay of high-intensity near work and environmental factors, the epidemiological characteristics of myopia have undergone significant changes: the age of onset of myopia has shown a clear trend of becoming younger (6–8). As a result, high myopia, which was previously mainly seen in older adolescents or adults [typically defined as spherical equivalent (SE) ≤ −6.00D)] (9), is now frequently observed in younger children and even preschoolers.

Unlike high myopia that develops in adulthood, high myopia that begins in childhood means that the patient will have a longer “exposure time” to myopia throughout their life. This early-onset, continuous eye expansion during the developmental period greatly increases the risk of irreversible pathological myopia in adulthood, including structural blinding complications such as posterior staphyloma, myopic macular degeneration, and retinal detachment (10, 11). Therefore, children and adolescents with high myopia are at extremely high risk, and there is an urgent need in clinical practice to establish a monitoring system for eye structure indicators that goes beyond simple refractive measurement to assess their potential long-term pathological risks and to conduct early monitoring and myopia prevention and control interventions.

At present, the clinical assessment of myopia progression still largely relies on changes in spherical equivalent (SE). However, a single SE value has significant limitations (12), as it is often influenced by compensatory mechanisms of the cornea or lens and cannot accurately reflect the substantial expansion of the posterior pole of the eye (13). In the group of children with high myopia, the clinical phenotype shows strong heterogeneity: not only may children with the same degree of myopia have completely different eye shapes, but also the onset of the disease in the left and right eyes of the same patient is often asynchronous, with significant refractive and structural asymmetry (14, 15). In addition, in outpatient visits, due to the limited cooperation of young children, irregular follow-up, and missing data records, it is particularly important to establish an eye structure evaluation index that is both scientifically sound and clinically operable. This is helpful for pediatric ophthalmologists to capture the true structural abnormal changes in the complex clinical manifestations.

Among the ocular biometric parameters, axial length (AL) is the most fundamental absolute indicator for assessing the progression of myopia. However, myopia is a manifestation of the mismatch between axial length and corneal curvature. For young children, their corneas are usually steeper and have a greater corneal curvature. Thus, merely focusing on axial length may underestimate the severity of myopia (16). In recent years, the axial ratio (AL/CR, the ratio of axial length to corneal curvature radius) has received extensive attention as a “structural ratio index” reflecting the overall abnormal morphology of the eyeball. AL/CR not only eliminates the interference of corneal refractive power but also helps to explain the degree of mechanical traction on the fundus (17). However, there is still a lack of clinical evidence regarding the distribution characteristics of AL/CR in low-age, early-onset high myopia children, its evolution with age, and its deep association with binocular asymmetry. Given the limitations of clinical retrospective data, this study does not aim to construct a definitive predictive model but rather incorporates AL/CR into routine assessment as an important exploratory structural metric, with the expectation of providing an ocular morphological perspective for evaluating and monitoring the progression of high myopia in children.

Therefore, this study aims to utilize the retrospective cohort data from our hospital’s outpatient department to explore the clinical and ocular structural characteristics of high myopia in children and adolescents: describe the distribution of baseline clinical and ocular biometric characteristics in the early-onset high myopia population, analyze the correlation between different ages and axial length (AL) and axial ratio (AL/CR), simultaneously quantify and evaluate binocular asymmetry (anisometropia), and explore its intrinsic connection with ocular structural differences. Finally, in the dataset of affected eyes with continuous follow-up records (≥2 times), describe the longitudinal progression trajectory and annual growth rate of ocular structural indicators in an exploratory manner.

## 2 Materials and methods

### 2.1 Study design and setting

This study adopted a single-center, retrospective, observational research design. Data collection was sourced from the Electronic Medical Record (EMR) system of the Pediatric Ophthalmology and Strabismus Refractive Error Clinic at the Second Affiliated Hospital of Dalian Medical University and the hospital’s outpatient ophthalmology examination data system. The data collection period covered all available consecutive visit records from January 1, 2024 to December 31, 2025.

### 2.2 Participants

**FIGURE 1:**
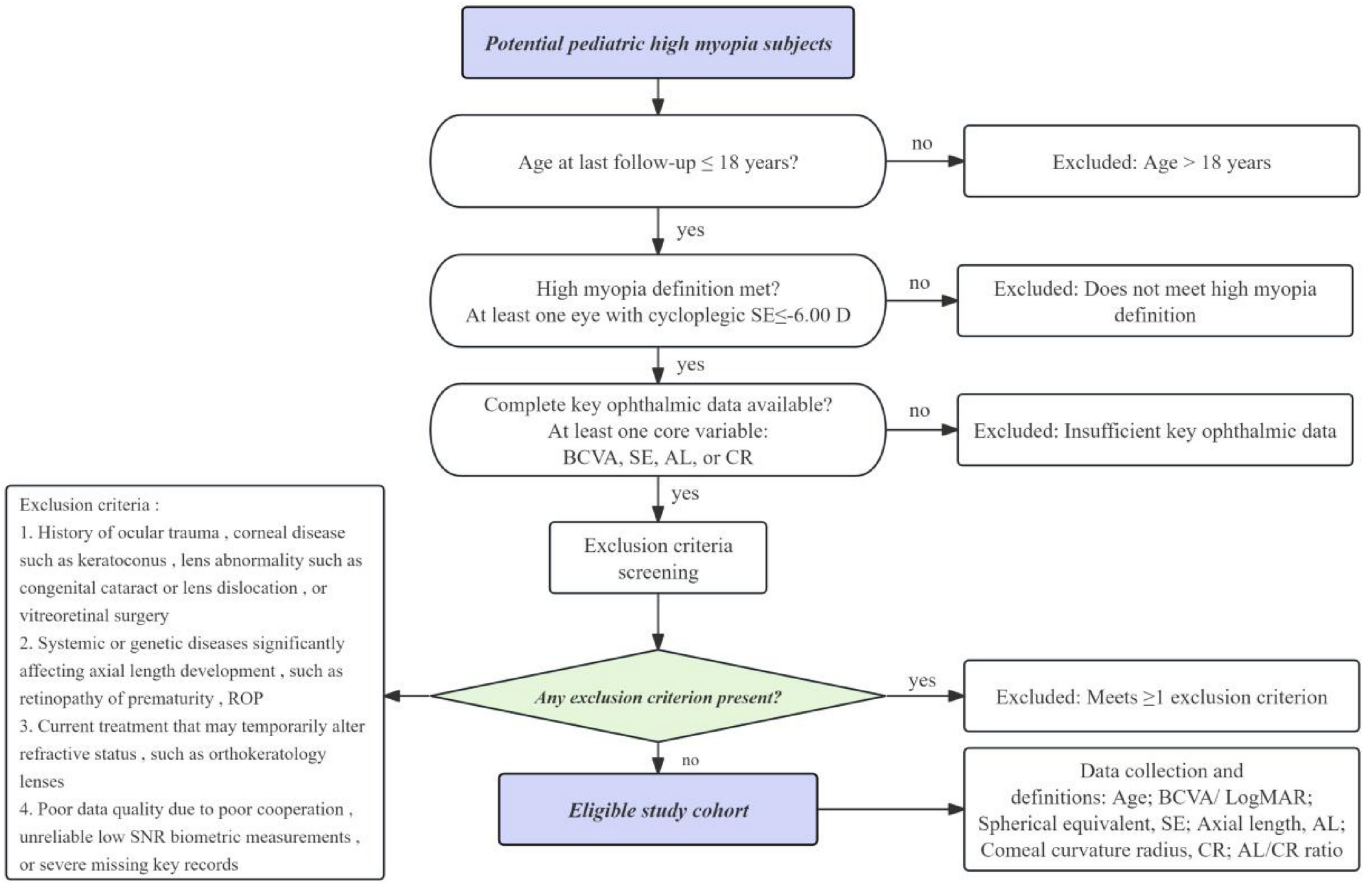
Study flow diagram of patient selection and exclusion criteria for the pediatric high myopia cohort. BCVA, best-corrected visual acuity; SE, spherical equivalent; AL, axial length; CR, corneal curvature radius.

The target population of this study was children and adolescents with high myopia who visited the hospital during the aforementioned period. The specific inclusion and exclusion criteria are as follows (see Figure 1 for details).

### 2.3 Inclusion

(1) The research subjects must meet both of the following conditions to be included: Age criterion: The age at the last follow-up is ≤ 18 years old (covering preschool children, school-age children, and adolescents); Definition of high myopia: At least one eye has a spherical equivalent (SE) of ≤ −6.00 D after cycloplegia.
(2) Data integrity: Complete key ophthalmic examination data are available, including at least one of the following core variables: best corrected visual acuity (BCVA), SE, axial length (AL), or corneal curvature radius (CR).

### 2.4 Exclusion

(1) Those with a history of ocular trauma, corneal diseases (such as keratoconus), lens abnormalities (such as congenital cataracts, lens dislocation), or a history of vitreoretinal surgery.
(2) Patients with systemic or genetic diseases: such as those with known systemic diseases that significantly affect axial length development, like retinopathy of prematurity (ROP).
(3) Those currently undergoing treatments that may temporarily alter refractive status, such as wearing orthokeratology lenses.
(4) Poor data quality: due to poor cooperation resulting in low, unreliable signal-to-noise ratio (SNR) of biometric measurements or severe missing of key records, making the data unsuitable for any statistical analysis.
(5) Data collection and definitions (Age, BCVA/LogMAR, SE, AL, AL/CR).

All clinical data were independently extracted and cross-checked by two trained ophthalmic researchers to ensure the accuracy of the data. The data we collected specifically included:

Demographic variables: The gender, date of birth, and examination date of the patients were recorded. Age (Age) was calculated as the interval from the date of birth to the examination date, and was precisely converted into a continuous variable (years) using the formula (examination date-date of birth)/365, with two decimal places retained, to replace the original “X years Y months” format and facilitate the correlation analysis of continuous variables.

Visual acuity: The best corrected visual acuity (BCVA) was originally recorded in the international standard decimal form. Before statistical analysis, all visual acuity data were converted to the logarithm of the minimum angle of resolution (LogMAR) visual acuity.

Refractive parameters: All children underwent standard cycloplegic refraction (dilation). The spherical equivalent (SE) was calculated using the formula: SE = Sphere+0.5 × Cylinder. High myopia was defined as SE ≤ −6.00D.

Ocular biometry: Axial length (AL) and corneal curvature radius (CR) were mainly obtained using the Lenstar optical interferometry axial length measurement instrument. For some young children who were difficult to cooperate, A-scan ultrasound was used. The axial ratio (AL/CR Ratio) was defined as the ratio of axial length to the average corneal curvature radius [CRmean = (CRflat + CRsteep)/2]. This ratio, as a parameter reflecting the shape of the eyeball, can eliminate the influence of corneal curvature on refraction and more directly reflect the degree of posterior pole expansion of the eyeball.

Interocular asymmetry: To quantify the differences in the development of both eyes, an absolute value index of interocular difference was introduced in this study. The interocular refractive difference was defined as | ΔSE| = | SE-Right-SE-Left|, and the interocular axial length difference was defined as | ΔAL| = | AL-Right-AL-Left| . Anisometropia, unless otherwise specified, was defined in this study as an interocular SE difference ≥ 2.00D.

Handling repeated visits and missing data.

As some patients had multiple follow-up records during the study period, to avoid selection bias and data correlation issues in statistics, the following stratified analysis strategy was adopted in this study:

Cross-sectional main analysis (Main Analysis): For the description of baseline characteristics, correlation analysis, and binocular asymmetry analysis, only the data from the last visit of each patient were included in the analysis. This time point represents the final ocular status of the patient at the end of the study and best reflects the structural changes after long-term myopia exposure.

Longitudinal Analysis: For the subset of patients with ≥ 2 valid biometric records during the study period and a follow-up interval of ≥ 6 months, they were included in the longitudinal analysis. The annual axial length elongation rate (AL Elongation Rate, mm/year) for each eye was calculated using a linear regression model, with the formula (last AL-first AL)/follow-up years, to dynamically assess the disease progression rate of the patients.

### 2.5 Statistical analysis

Data analyses were conducted using SPSS 26.0.

(1) For descriptive statistics, continuous variables were first tested for normality. Data conforming to a normal distribution were expressed as mean ± standard deviation (Mean ± SD), while data not following a normal distribution were presented as median (interquartile range, IQR). Categorical variables were expressed as frequency (percentage, %).
(2) Correlation analysis: Different statistical methods were selected based on whether the dataset was normally distributed. Pearson correlation coefficient (for normal distribution) or Spearman rank correlation coefficient (for non-normal distribution) was used to evaluate the linear relationship between age and AL, AL/CR, as well as the correlation between binocular refractive difference and structural difference (| ΔSE| and | ΔAL|).
(3) Differences were compared between groups (such as unilateral high myopia versus bilateral high myopia) using independent samples *t*-test or Mann-Whitney U test. Comparisons of categorical variables were conducted using Chi-square test or Fisher’s exact test.
(4) Sensitivity analysis: Given the strong biological correlation (inter-eye correlation) between the left and right eyes of the same patient, treating them as independent samples directly may lead to an increase in Type I error.
(5) GEE statistical analysis: To account for the inherent correlation between the two eyes of the same patient, Generalized Estimating Equations (GEE) with an exchangeable working correlation structure were employed to examine the associations between age and ocular biometric parameters (AL and AL/CR), as well as between refractive severity (SE) and AL after adjusting for age. Separate GEE models were constructed with the following dependent variables and predictors: ➀AL ∼ Age; ➁AL/CR ∼ Age; ➂AL ∼ Age + Sex; ➃AL ∼ SE + Age; and ➀BCVA (LogMAR) ∼ AL. A Gaussian family with identity link function was specified for all models. Robust (Huber-White) sandwich variance estimators were used to compute standard errors. Binocular asymmetry was assessed using Spearman’s rank correlation between | ΔSE| and | ΔAL| at the patient level (*n* = 65 patients with complete bilateral AL data). All statistical tests were two-sided, and *P* < 0.05 were considered statistically significant. Analyses were performed using the statsmodels package (v0.14) in Python 3.12.

All statistical tests were two-sided, and *P* < 0.05 was considered statistically significant.

### 2.6 Ethics

The study was approved by the Ethics Committee of the Second Affiliated Hospital of Dalian Medical University (Approval Number: KY2025-182-01) and strictly adhered to the ethical principles for medical research involving human subjects as stipulated in the Declaration of Helsinki. All the parents of the patients signed the informed consent forms.

This study strictly adheres to the principle of patient privacy protection. During the data extraction and cleaning process, all fields containing personal identity information of patients (such as names and outpatient numbers) have been removed or anonymized through de-identification. The research only uses necessary clinical data for scientific analysis, and will not interfere with patients’ treatment decisions or increase any additional risks for them.

## 3 Results

### 3.1 Study population and baseline characteristics

In this study, a total of 84 patients under the age of 18 (mean age 11.64 ± 3.85 years, range 2.17–17.00 years) met the inclusion criteria, including 47 males (56.0%) and 37 females (44.0%). Overall, 62 patients (73.8%) had high myopia in both eyes, and 22 (26.2%) had high myopia in one eye, involving a total of 146 high myopia eyes (spherical equivalent ≤ −6.00D). Among the high myopia eyes, the mean spherical equivalent was −8.87 ± 2.58D (median −8.38D, interquartile range: −10.25 to −7.00). The mean axial length (AL) was 26.41 ± 1.33 mm (median 26.33 mm, interquartile range: 25.55–27.21), and axial length data were available for 118 eyes (80.8%) out of 146. The ratio of axial length to corneal radius (AL/CR) was available for 73 eyes (50.0%), with a mean of 3.38 ± 0.19 (median 3.38, interquartile range 3.29–3.49). Best corrected visual acuity (BCVA) converted to LogMAR was available for 136 eyes (93.2%), with a mean of 0.20 ± 0.31 (median 0.10, interquartile range: 0.00–0.22) (see Table 1 for details).

**TABLE 1:** Baseline demographic and ocular characteristics at the last visit in pediatric/adolescent high-myopia cohort.

Characteristic	n available	Mean ± SD or *n*(%)	Median (IQR)	Missing *n*(%)
Patient-level (patients with ≥ 1 high-myopic eye)				
Number of patients	84	–	–	0(0.0)
Age at last visit, years	84	11.64 ± 3.85	12.00(9.15,15.0)	0(0.0)
Sex				
Male	84	47(56.0%)	–	0(0.0)
Female	84	37(44.0%)	–	0(0.0)
Laterality of high myopia (by eye, at last visit)				
Bilateral high myopia	84	62(73.8%)	–	0(0.0)
Unilateral high myopia	84	22(26.2%)	–	0(0.0)
Eye-level (high-myopic eyes only, SE ≤ -6.00 D)				
Number of high-myopic eyes	146	–	–	0(0.0)
BCVA, LogMAR	136	0.20 ± 0.31	0.10(0.00,0.22)	10(6.8)
Spherical equivalent (SE), D	146	−8.87 ± 2.58	−8.38(−10.25,−7.00)	0(0.0)
Axial length (AL), mm	118	26.41 ± 1.33	26.33(25.55,27.21)	28(19.2)
AL/CR ratio	73	3.38 ± 0.19	3.38(3.29,3.49)	73(50.0)

Definition of high myopia: At the last follow-up, SE ≤ -6.00D (determined by eye). BCVA is converted from decimal visual acuity to LogMAR (the larger the LogMAR, the worse the visual acuity; LogMAR = 0 corresponds to BCVA = 1.0). Missing values indicate that the index was not recorded at the last follow-up. All continuous variable statistics are complete-case (calculated using available samples).

### 3.2 Age-related trends in AL and AL/CR

**FIGURE 2:**
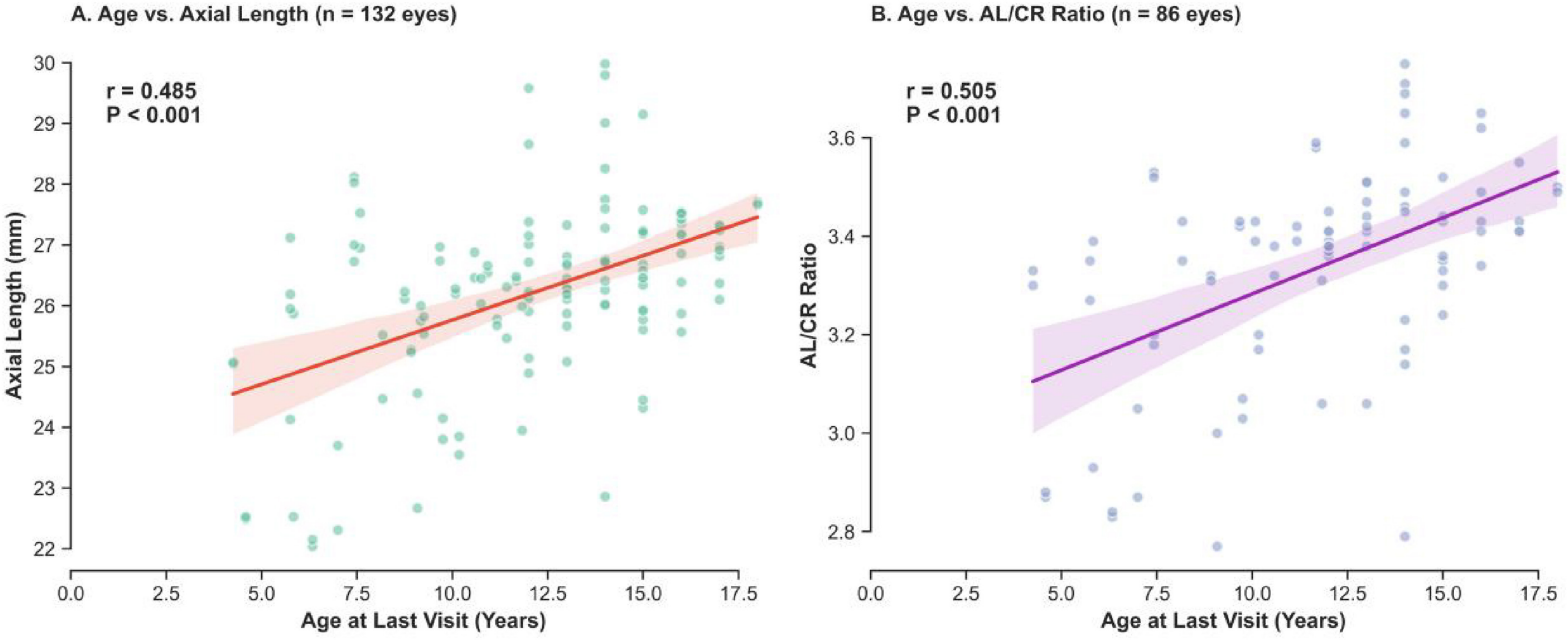
Cross-sectional associations between age and ocular biometrics at the last visit in the pediatric/adolescent high-myopia cohort. Scatter plots with linear regression lines demonstrate the relationships between chronological age and (A) axial length (AL) in 132 individual eyes, and (B) the axial length-to-corneal radius (AL/CR) ratio in 86 individual eyes. The shaded areas represent the 95% confidence intervals of the regression models. Both structural parameters exhibit significant positive correlations with age (Pearson *r* = 0.485 and *r* = 0.505, respectively; both *P* < 0.001), indicating ongoing, uncompensated axial elongation as the pediatric patients age.

To assess the structural changes of high myopia in children during their physical development period, we first conducted a cross-sectional analysis of the relationship between age and key ocular biological parameters (Figure 2). The scatter plots and the fitted linear regression models visually demonstrated the ongoing expansion of the eyeball. As shown in Figure 2A, among 132 eyes, although all patients had reached the refractive threshold of high myopia, the axial length (AL) of the eyes did not stabilize but showed a highly significant positive linear correlation with age (Pearson *r* = 0.485, *P* < 0.001). This indicates that the axial length of older children is generally longer than that of younger children.

To further eliminate the interference caused by the overall proportional enlargement of the eyeball (scaling effect), we introduced a core indicator reflecting the disproportionate structure of the eyeball − the AL/CR ratio (Figure 2B). In 86 eyes, the AL/CR ratio also showed a significant upward trend with age (Pearson *r* = 0.505, *P* < 0.001). The scatter distribution characteristics indicated that as age advanced, a considerable number of eyes exceeded the threshold limit representing significant structural deformation. This finding strongly confirmed that in the population of children with high myopia, the compensatory capacity of the anterior corneal segment has been exhausted, and the elongation of the eyeball is essentially an unbalanced pathological expansion process at the posterior pole.

### 3.3 Generalized estimating equations (GEE) analysis of ocular biometric patterns

To further evaluate the longitudinal association between age and ocular biometric parameters while accounting for within-subject inter-eye correlation, we performed Generalized Estimating Equations (GEE) analyses with an exchangeable correlation structure.

**FIGURE 3:**
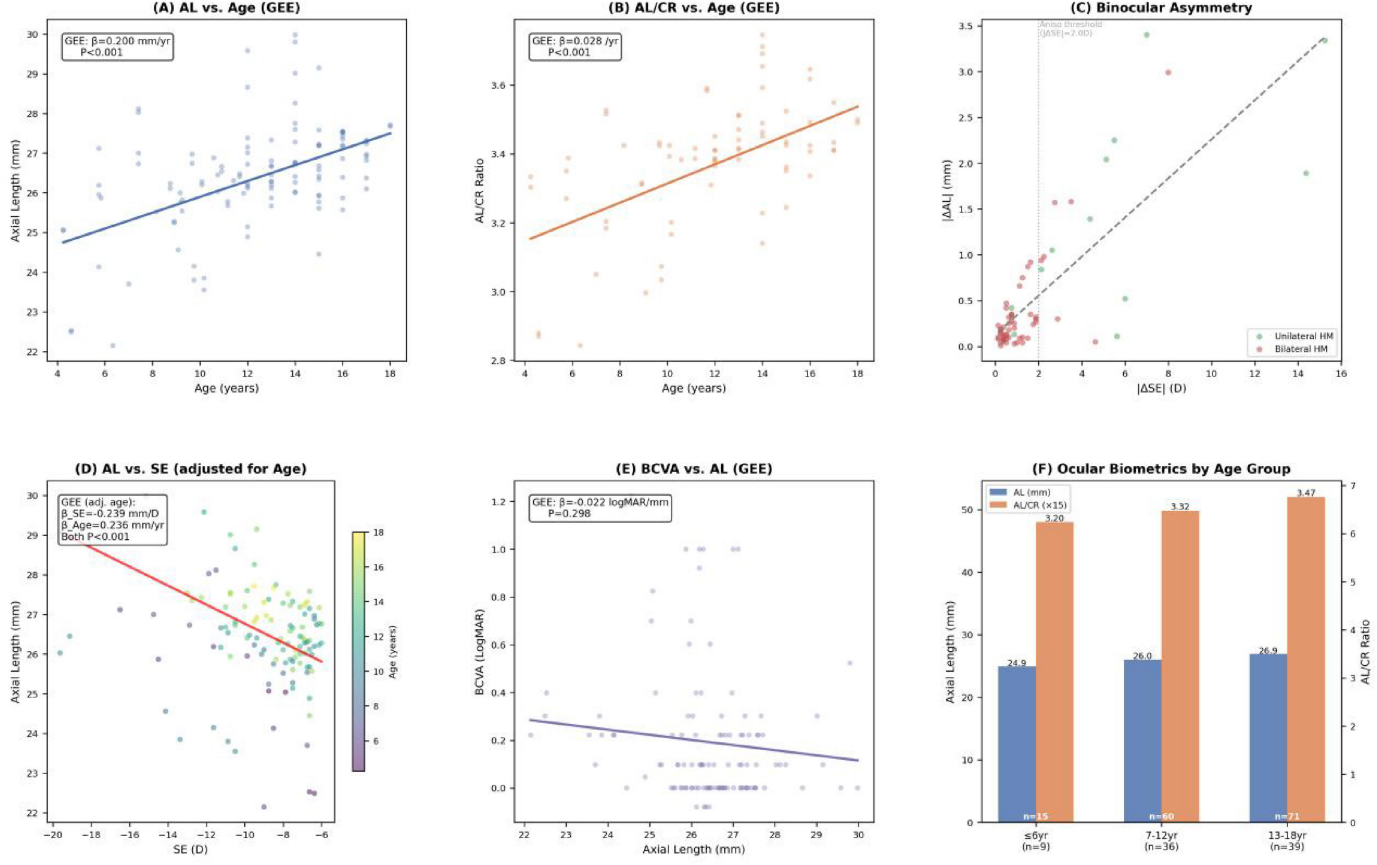
Generalized estimating equations (GEE) analysis of ocular biometric patterns in pediatric high myopia. **(A)** Association between age and axial length (AL). Scatter plot with GEE-derived linear regression line (β = 0.200 mm/year, *P* < 0.001). Each point represents one highly myopic eye. **(B)** Association between age and axial length/corneal radius ratio (AL/CR) (β = 0.028 per year, *P* < 0.001). **(C)** Relationship between binocular refractive asymmetry (| ΔSE|) and structural asymmetry (| ΔAL|). Blue: unilateral high myopia (*n* = 13); orange: bilateral high myopia (*n* = 52). Dashed vertical line indicates clinical anisometropia threshold (| ΔSE| = 2.0 D). Gray dashed line: linear trend. Spearman ρ = 0.629, *P* < 0.001. **(D)** Scatter plot of axial length against spherical equivalent (SE), color-coded by age. GEE regression line at median age shows the independent effect of SE on AL after adjusting for age (β_SE = −0.239 mm/D, *P* < 0.001). **(E)** Best-corrected visual acuity (LogMAR) vs. axial length. GEE regression line (β = −0.022 logMAR/mm, *P* = 0.298) indicates no statistically significant association. **(F)** Grouped bar chart comparing mean AL and AL/CR across three age groups ( ≤ 6, 7–12, 13–18 years). AL/CR values scaled by × 15 for visual comparison. Eye counts per group displayed within bars. Error bars represent ± 1 SD. All GEE models used exchangeable working correlation structure. *P* < 0.05 was considered statistically significant.

As shown in Figure 3 and Table 2, age was significantly positively associated with axial length (AL) (β = 0.200 mm/year, SE = 0.046, 95% CI: 0.109 to 0.291, *P* < 0.001; Figure 3A). Similarly, the AL/corneal radius ratio (AL/CR) demonstrated a significant positive association with age (β = 0.028 per year, SE = 0.008, 95% CI: 0.013–0.043, *P* = 0.0003; Figure 3B), confirming that both absolute axial elongation and structural disproportionality progress with age in pediatric high myopia. After adjusting for age, SE remained significantly associated with AL, with each 1.00D increase in myopia corresponding to a 0.239 mm increase in axial length (β = −0.239 mm/D, SE = 0.052, 95% CI: −0.341 to −0.138, *P* < 0.001; Figure 3D), indicating that refractive severity is an independent predictor of ocular structural elongation beyond age-related changes. Sex was not a significant predictor when added to the model (*P* = 0.465).

**TABLE 2:** Generalized estimating equations (GEE) analysis of ocular biometric parameters in pediatric high myopia.

Model	Predictor	β	SE	95% CI	*P*	N (eyes)	*n* (patients)
1	Age → AL (mm/yr)	0.200	0.046	(0.109, 0.291)	<0.001	116	65
2	Age → AL/CR (per yr)	0.028	0.008	(0.013, 0.043)	<0.001	73	43
3	Age → AL (Sex-adjusted, mm/yr)	0.203	0.046	(0.113, 0.294)	<0.001	116	65
4a	SE → AL (Age-adjusted, mm/D)	−0.239	0.052	(−0.341, −0.138)	<0.001	116	65
4b	Age → AL (SE-adjusted, mm/yr)	0.236	0.048	(0.142, 0.331)	<0.001	116	65
5	| ΔSE| ↔ | ΔAL| (Spearman)	ρ = 0.629	–	–	<0.001	65^1^	65
6	AL → BCVA (logMAR/mm)	−0.022	0.021	(−0.062, 0.019)	0.298	113	63

^1^Patient-level analysis (patients with complete bilateral AL data). GEE, generalized estimating equations with exchangeable correlation structure; SE, standard error; AL, axial length; AL/CR, axial length/corneal radius ratio; BCVA, best-corrected visual acuity.

Binocular asymmetry analysis revealed a strong positive correlation between the absolute interocular difference in spherical equivalent (| ΔSE|) and the absolute difference in axial length (| ΔAL|) (Spearman’s ρ = 0.629, *P* < 0.001; Figure 3C). This robust association persisted across both bilateral and unilateral high myopia subgroups. Notably, the Spearman coefficient was nearly identical whether calculated within the patient-level dataset [ρ = 0.629 (*n* = 65)] or combined eye-level GEE framework (Pearson *r* = 0.788, *P* < 0.001), underscoring that greater binocular refractive disparity consistently predicts greater structural asymmetry. Best-corrected visual acuity (BCVA, LogMAR) was not significantly associated with axial length (β = −0.022 logMAR/mm, SE = 0.021, 95% CI: −0.062 to 0.019, *P* = 0.298; Figure 3E), suggesting that in pediatric high myopia, axial elongation alone does not directly determine the degree of visual impairment, and other factors (e.g., amblyopia, anisometropia) may play a more prominent role. Figure 3F summarizes the distribution of AL and AL/CR across three age groups (≤6, 7–12, and 13–18 years), demonstrating a consistent increase in both parameters with advancing age. These findings collectively highlight the value of AL and AL/CR as dynamic structural biomarkers for monitoring progression and risk stratification in pediatric high myopia management.

### 3.4 Interocular asymmetry

This study evaluated the binocular asymmetry at the last follow-up and compared the differences between patients with bilateral high myopia (*n* = 62) and those with unilateral high myopia (*n* = 22) (Table 3). Overall, the median of the absolute spherical equivalent difference (| ΔSE|) was 0.88D. Notably, the degree of refractive asymmetry in patients with unilateral high myopia was significantly higher than that in patients with bilateral high myopia (median | ΔSE| : 4.37D vs. 0.81D, *P* < 0.001). Therefore, the prevalence of clinically significant anisometropia (| ΔSE| ≥ 2.0D) in the unilateral high myopia group was also significantly higher than that in the bilateral group (66.7% vs. 14.5%, *P* < 0.001).

**TABLE 3:** Interocular asymmetry at the last visit and comparison between bilateral and unilateral high myopia.

Variables	Overall cohort (*N* = 84)	Bilateral high myopia (*n* = 62)	Unilateral high myopia (*n* = 22)	*P-*value*
Interocular SE difference (| ΔSE|), D	2.11 ± 2.95 [0.88 (0.50–2.19)] (*n* = 83)	1.17 ± 1.29[0.81 (0.37–1.50)]	4.89 ± 4.46[4.37 (1.13–6.00)] (*n* = 21)	<0.001
Anisometropia (| ΔSE| ≥ 2.0D), n/N (%)	23/83(27.7%)	9/62(14.5%)	14/21(66.7%)	<0.001
Interocular AL difference (| ΔAL|), mm	0.56 ± 0.78[0.25 (0.10–0.64)] (*n* = 66)	0.37 ± 0.48[0.22 (0.10–0.45)] (*n* = 52)	1.27 ± 1.25[0.95 (0.31–1.95)] (*n* = 14)	0.002
Interocular LogMAR difference	0.16 ± 0.30[0.08 (0.00–0.12)] (*n* = 77)	0.14 ± 0.29[0.04 (0.00–0.12)] (*n* = 58)	0.24 ± 0.32[0.10 (0.00–0.24)] (*n* = 19)	0.128

| Δ| represents the absolute difference between both eyes at the last follow-up (e.g., | ΔSE| = | SE_R − SE_L|). Bilateral high myopia: both eyes meet SE ≤ −6.00D at the last follow-up; unilateral high myopia: only one eye meets this criterion. For comparisons between groups of continuous variables: Mann–Whitney U test; for categorical variables: Fisher’s exact test. The Spearman ρ of | ΔSE| and | ΔAL| was 0.629, *P* < 0.001 (*n* = 66).

**FIGURE 4:**
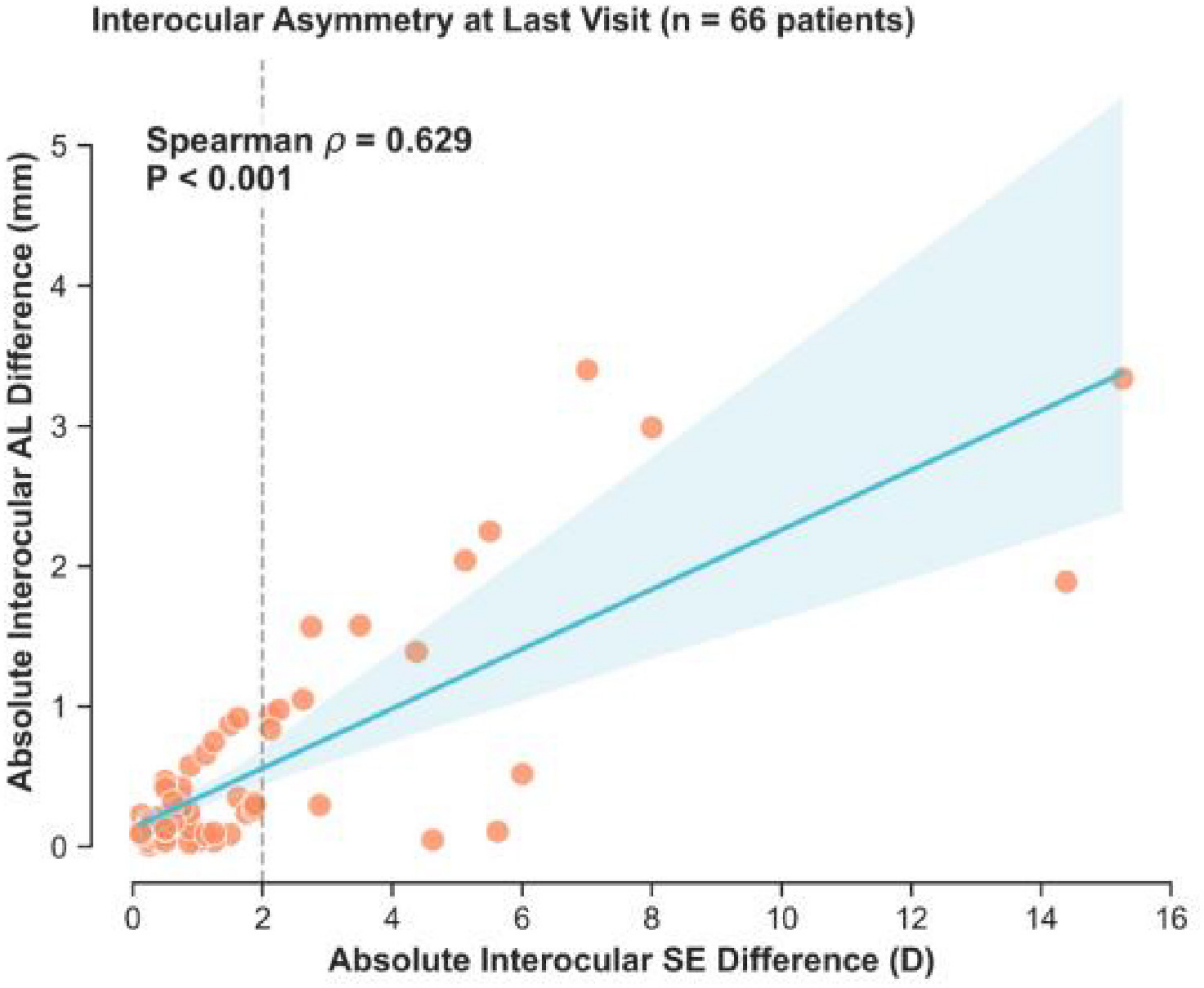
Correlation between refractive and structural interocular asymmetry at the last visit. The scatter plot visualizes the strong positive relationship between the absolute interocular difference in spherical equivalent (—ΔSE—) and the absolute interocular difference in axial length (—ΔAL—) among 66 patients. The vertical dashed line demarcates the clinical threshold for anisometropia (—ΔSE— ≥ 2.0D). Given the non-normal distribution of the asymmetry data, a robust non-parametric test was applied, revealing a highly significant correlation (Spearman’s ρ = 0.629, *P* < 0.001).

Structural asymmetry also showed a similar trend. The absolute axial length difference (| ΔAL|) in the monocular high myopia group was significantly greater than that in the binocular high myopia group (median 0.95 mm vs. 0.22 mm, *P* = 0.002). A correlation analysis of 66 patients with complete binocular axial length data further confirmed that anisometropia was mainly caused by axial length asymmetry. That is, there was a strong positive correlation between | ΔSE| and | ΔAL| (Spearman ρ = 0.629, *P* < 0.001; see Figure 4).

Despite the significant binocular differences in structure and refraction between the two groups, there was no statistically significant difference in the absolute difference of best-corrected visual acuity (| ΔLogMAR|) between the two groups (median 0.10 vs. 0.04, *P* = 0.128). This indicates that during the follow-up period, severe structural asymmetry did not cause disproportionate damage to the visual outcomes of the high myopia group with unilateral eyes.

### 3.5 Exploratory longitudinal observation

**FIGURE 5:**
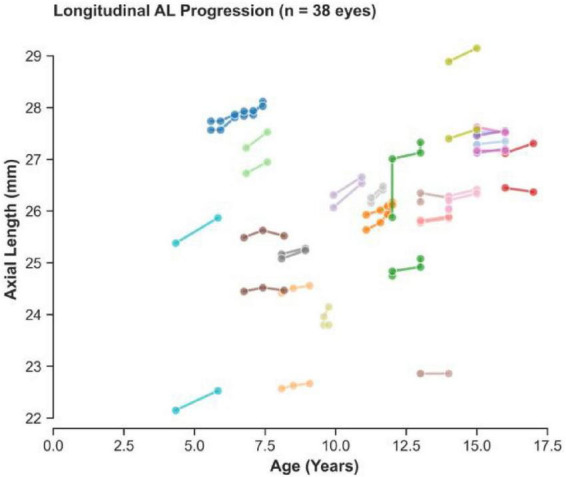
Longitudinal trajectories of axial elongation during the pediatric follow-up period. Unlike the cross-sectional analyses at the last visit, this spaghetti plot depicts the dynamic, real-world progression of axial length over time in a subset of 38 eyes with repeated biometric measurements at distinct ages. Each connected line segment tracks the longitudinal trajectory of a single eye. The prevailing upward slopes visually confirm that active structural expansion persists without spontaneous stabilization during the observation window in this high-myopia cohort.

The cross-sectional correlation analysis shown in Figure 2 provides static evidence for the developmental elongation of the eyeball, while the longitudinal follow-up subset (*n* = 38 eyes) we established in Figure 5 offers dynamic confirmation of this process. Among the eyes with longitudinal tracking data (Figure 5), a total of 34 eyes had an effective follow-up period of at least 6 months (average follow-up time: 1.07 years). During this period, the highly myopic cohort of children showed continuous structural expansion. The overall average annual axial length increase was as high as 0.224 ± 0.264 mm/year (Median: 0.190 mm/year), with the fastest progressing eye reaching 1.450 mm/year.

This spaghetti plot depicts the independent growth trajectories of each eye after different follow-up periods. It can be visually observed that the majority of the single-eye trajectories show a distinct upward slope direction (positive sloped pathways). Even in cases where the axial length of the eye has reached extremely high levels (such as > 26.5 mm), the lines still do not show the expected flat and broad “plateau” phase. On the contrary, the structural progression of these affected eyes remains active and even accelerates. Longitudinal follow-up data indicate that high myopia in children is difficult to stabilize naturally before the age of 18, emphasizing the necessity of strict biometric monitoring and myopia control intervention during this critical window period.

## 4 Discussion

This study collected and analyzed the clinical data of pediatric ophthalmology outpatient services in our hospital to systematically describe the binocular asymmetry and ocular biometric parameters of high myopia in children and adolescents. The results showed that the axial length and AL/CR levels of the children were at a relatively high level, indicating that significant changes had occurred in their ocular structure. Additionally, this study observed a positive correlation between age and axial length, which is consistent with the basic laws of visual development in children and adolescents. During childhood, the eye is still in a growth and development window, and under normal circumstances, the axial length will increase slowly with age. Relevant consensus suggests that an increase of less than 0.20 mm/year in axial length is considered a safe growth range for children and adolescents aged 6–10 years, while for children under 6 years old, the safe growth range of axial length should be higher than this threshold, and for those over 10 years old, the safe annual growth of axial length should be lower than this threshold (18). One study recommended that an AL/CR ratio greater than 3.00 in 9-year-old children be used as the threshold for diagnosing myopia, and a value above this diagnostic threshold indicates a higher risk of myopia and a possible depletion of the far-sighted reserve (19). In children with high myopia, the axial length grows at a faster rate. In the longitudinal follow-up data of this study, the average annual increase in axial length was as high as 0.224 ± 0.264 mm/year, with the fastest-growing eye reaching 1.450 mm/year. This result still supports the widely accepted view in clinical practice that high myopia in children is not a fixed refractive state but a process that continuously changes over time. The younger the child, the lower the biomechanical strength of the sclera, and the greater the space for axial length growth, thus the longer the potential risk exposure time. For clinicians, this indicates that we should not rely solely on a single refraction result to determine the stability of the condition, but should use axial length as an important indicator for dynamic monitoring, especially for children with early-onset high myopia, who require long-term and continuous assessment of their structural changes.

It is worth noting that in this cohort, AL/CR was strongly associated with poor corrected visual acuity (increased LogMAR), suggesting that AL/CR may indicate the risk of “restricted vision after correction” due to ocular structural abnormalities. The results of this study support the use of AL/CR as a supplementary indicator: corneal curvature can compensate for SE to a certain extent. If only refractive error or AL is used for assessment, the posterior pole expansion of the eye may be underestimated. AL/CR, on the other hand, can sensitively reflect the deviation of ocular morphology and the potential vulnerability of the sclera in terms of biomechanics. Therefore, high myopia in childhood should be regarded as an “early high-risk state of continuous structural changes.” In younger children who are still in the rapid axial elongation window, the increase in AL and AL/CR indicates a longer exposure to myopia and a higher cumulative risk of pathological myopia, which has value in risk stratification.

In addition, anisometropia is relatively common in children with high myopia. Especially, in some children, the difference in equivalent spherical power between the two eyes reaches 2.0D or more, which indicates that we should not ignore the issue of anisometropia in children with high myopia in clinical practice. In the scatter plot in Figure 4, we can clearly observe that most data points are closely clustered around the regression line. The gray dotted line marks the clinical alert line of | ΔSE| ≥ 2.0D for anisometropia. As the | ΔAL| distance increases, the difference scatter points steadily shift to the right high anisometropia area. This means that in children with high myopia, when the myopia degree of one eye is significantly higher than that of the contralateral eye, the main cause of this difference is the abnormal excessive elongation of the eye axis of that eye, rather than changes in corneal or lens refractive power. That is, the anisometropia in this group of patients is characterized by typical “axial anisometropia” (Axial anisometropia), and related studies have also confirmed this (20–22). This finding is of great significance for clinical follow-up. For children with unilateral high myopia or significant interocular differences, more attention should be paid to the development imbalance between the dominant eye and the non-dominant eye. This asymmetry may indicate that the myopia progression rate of the unilateral eye is faster and the structural risk is higher. At the same time, anisometropia may also increase the risk of amblyopia, binocular visual function abnormalities, and future visual development problems (23). Therefore, such children should undergo more detailed evaluations in the outpatient clinic, including visual acuity, refractive status, axial length, fundus condition, and binocular visual function, etc. Overall, binocular asymmetry should not be regarded as simply “two eyes being different,” but should be seen as an important manifestation of the heterogeneity of high myopia in children and an important basis for individualized follow-up.

To further account for the inherent correlation between the two eyes of the same patient, we employed Generalized Estimating Equations (GEE) with an exchangeable working correlation structure. This analytical approach allows each eye to contribute information while appropriately adjusting the standard errors for within-subject clustering, thereby providing more robust and reliable estimates of the associations between ocular parameters (Figure 3 and Table 2). The GEE results corroborated the primary findings from the conventional Pearson correlation analysis: age remained a significant predictor of both AL (β = 0.200 mm/year, 95% CI: 0.109–0.291, *P* < 0.001) and AL/CR (β = 0.028 per year, 95% CI: 0.013–0.043, *P* < 0.001) after adjusting for inter-eye correlation. The similarity between the Pearson correlation coefficients (*r* = 0.485 for AL–age; *r* = 0.505 for AL/CR–age) and the GEE regression slopes suggests that inter-eye correlation did not substantially distort the cross-sectional associations in this cohort.

Importantly, the GEE model simultaneously incorporating both SE and age revealed that the effect of refractive error on axial length remained significant after adjusting for age (β_SE = −0.239 mm/D, 95% CI: −0.341 to −0.138, *P* < 0.001), while the age effect also persisted after accounting for SE (β_age = 0.236 mm/year, 95% CI: 0.142–0.331, *P* < 0.001). This confirms that in pediatric high myopia, axial elongation is jointly driven by both developmental growth (age) and the severity of the refractive error itself—two factors that exert independent and additive effects on ocular structure. Furthermore, the Spearman correlation between binocular refractive asymmetry (| ΔSE|) and structural asymmetry (| ΔAL|) was moderate to strong (ρ = 0.629, *P* < 0.001), supporting that anisometropia in this population is predominantly axial in nature, consistent with the concept of “axial anisometropia” discussed above. In contrast, the GEE analysis of BCVA against AL showed no statistically significant association (β = −0.022 logMAR/mm, *P* = 0.298), suggesting that within the range of axial lengths observed in this cohort, the degree of axial elongation alone does not linearly predict best-corrected visual acuity loss. This finding underscores the multifactorial nature of visual function impairment in pediatric high myopia, in which factors such as macular morphology, choroidal thickness, and amblyogenic risk may play a more determinant role than absolute axial length alone.

In this study, some children still had a visual acuity of only 0.1–0.4 even after the best possible correction, suggesting that early-onset high myopia not only affects refractive status but may also have a substantial impact on visual function. We speculate that the extremely high AL/CR ratio reflects excessive axial elongation of the eyeball, which may cause long-term traction on the macular retina and choroid, thereby affecting the density of receptor cells and the integrity of retinal microstructure. Relevant studies have confirmed that the choroidal thickness in patients with high myopia also thins (24). For younger children with earlier onset of the disease, such structural changes may occur during the critical period of visual development, so even with appropriate glasses, the best corrected visual acuity is still difficult to reach the normal level, showing a certain degree of “organic visual impairment” characteristics. On the other hand, children with significant binocular asymmetry are more likely to have unbalanced visual function development, especially those with unilateral high myopia or large anisometropia, and the possibility of amblyopia and binocular vision abnormalities should be vigilant. This also indicates that the management of high myopia in children should not only focus on the degree itself but also make a comprehensive judgment based on visual function and structural indicators. For young children, the earlier the abnormal growth of the eye axis and binocular asymmetry are detected, the more likely it is to reduce subsequent visual function damage through early intervention.

From a clinical perspective, this study suggests that pediatric ophthalmologists should not rely solely on spherical equivalent to assess the risk of high myopia in their outpatient clinics. Instead, they should incorporate axial length, AL/CR, and interocular asymmetry into their routine assessment system for comprehensive analysis. Especially for younger children with significantly elevated AL/CR, the frequency of follow-up should be increased to identify high-risk individuals with rapid structural progression as early as possible and take more proactive myopia control measures as needed. At the same time, for children with unilateral high myopia or significant interocular differences, the risk of amblyopia, visual function status, and fundus changes should also be evaluated simultaneously to achieve more refined management. From a public health perspective, this structured monitoring approach helps identify children who truly need focused management, improving the efficiency of resource allocation. In cases of extreme progression and poor response to conventional optical and pharmacological control, the possibility of procedures such as posterior scleral reinforcement surgery can be discussed on the basis of strict indications and specialized assessment, but the risks and benefits must be carefully weighed.

This study also has some limitations. First, this was a retrospective, single-center, clinic-based study; inclusion of patients who presented for care may introduce selection bias and limits generalizability to the broader pediatric population. Second, the sample size—particularly for unilateral high myopia and for longitudinal follow-up—was modest, and the estimation of axial elongation rates should therefore be interpreted as exploratory. Third, AL/CR ratio was unavailable for a substantial proportion of eyes because keratometry was not routinely recorded for all visits in this real-world dataset; we performed complete-case analyses for AL/CR-related models and cannot fully exclude residual bias due to missing data. Fourth, information on myopia-control interventions (e.g., atropine, orthokeratology, and myopia-control spectacles/contact lenses) and key environmental exposures (near work, outdoor time, and screen use) was not consistently documented, precluding adjustment for these potential confounders. Fifth, the absence of multimodal fundus/OCT biomarkers, choroidal metrics, and genetic data limited mechanistic inference and the ability to directly link biometric risk to early pathologic changes. Finally, BCVA assessment in younger children may be influenced by cooperation and testing conditions, and thus structure–visual acuity associations should be interpreted cautiously. Future multicenter prospective cohorts with standardized biometric and imaging protocols and longer follow-up are needed to validate these findings.

## 5 Conclusion

In summary, high myopia in children and adolescents is characterized by continuous structural expansion of the eyeball. It is worth noting that unilateral high myopia is a type that is more likely to damage binocular visual function. Due to the unequal growth of the ocular axes of both eyes, it shows a mismatch in refractive power between the two eyes. Despite the structural asymmetry of both eyes, during the developmental stage of childhood and adolescence, there is no significant difference in corrected visual acuity between the two eyes, which provides a crucial time window for clinical intervention. Our research has confirmed the urgency of early monitoring and active intervention of ocular biological parameters such as axial length and axial ratio in children and adolescents. At the same time, this is crucial for preventing high myopia-induced amblyopia and irreversible pathological changes in the fundus.

## Data Availability

The original contributions presented in this study are included in the article/supplementary material, further inquiries can be directed to the corresponding author.
